# Supplementary material for: UCHL5 Promotes Proliferation and Migration of Bladder Cancer Cells by Activating c-Myc via AKT/mTOR Signaling
Source: Cancers (Basel). 2022 Nov 10;14(22):5538. doi: 10.3390/cancers14225538 (PMC9688806; doi:10.3390/cancers14225538)

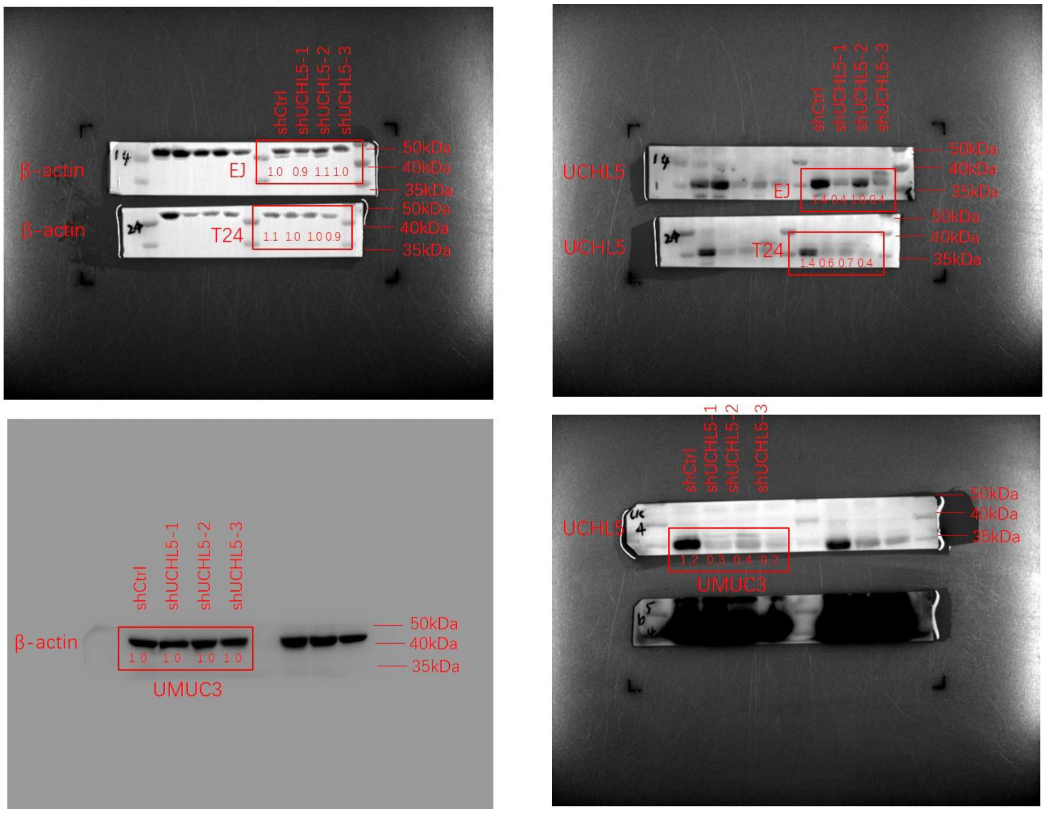


Figure 2A：Original pictures of western blot bands including intensity ratio

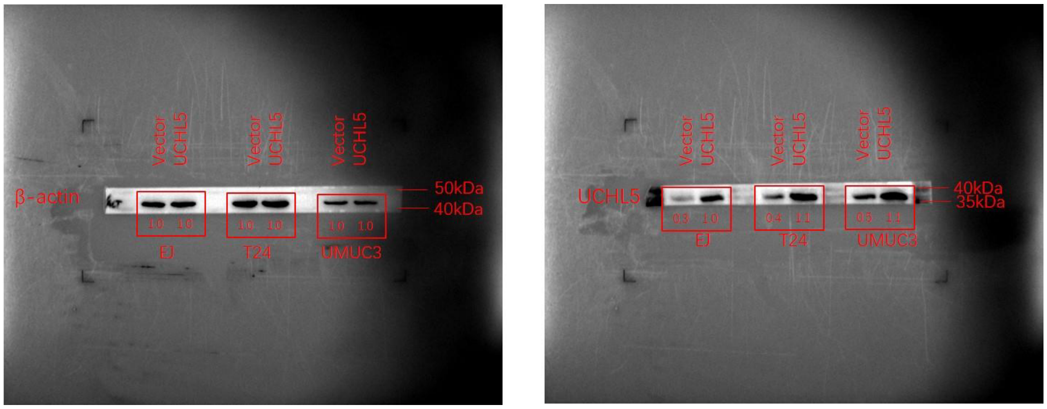


Figure 3A：Original pictures of western blot bands including intensity ratio

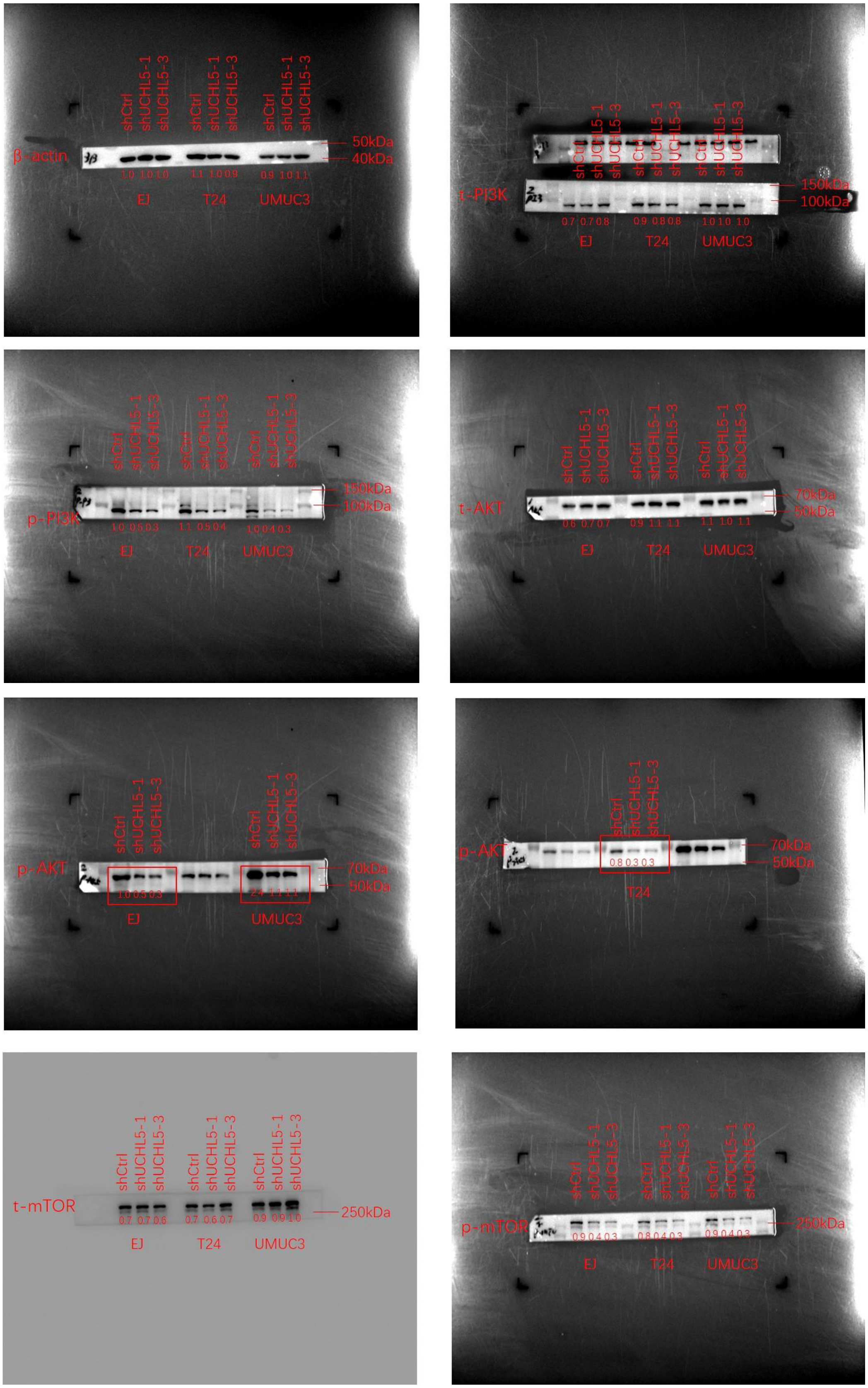


Figure 4H：Original pictures of western blot bands including intensity ratio

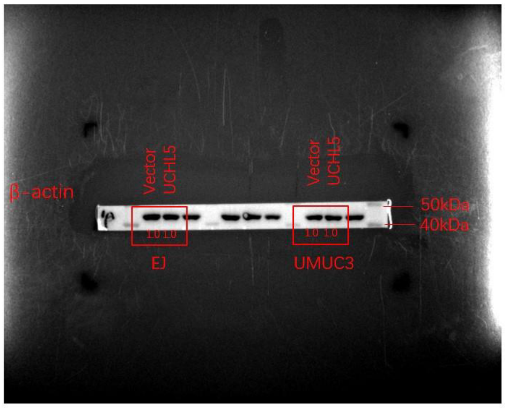
β-actin
Vector
UCHL5
Vector
UCHL5
50kDa
40kDa
EJ
UMUC3

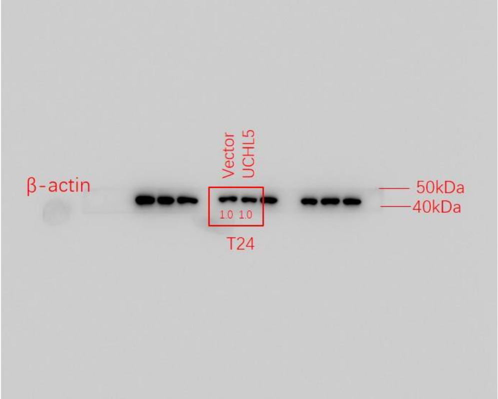
β-actin
Vector
UCHL5
50kDa
40kDa
T24

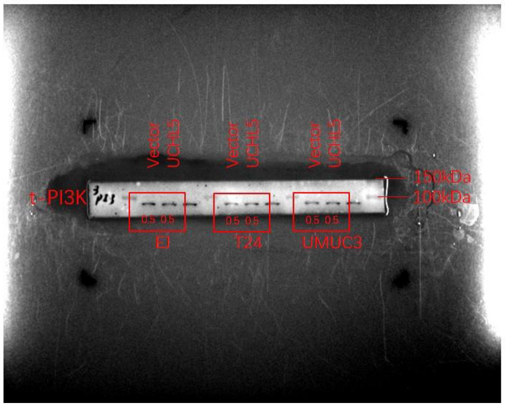
t-PI3K
Vector
UCHL5
Vector
UCHL5
Vector
UCHL5
150kDa
100kDa
EJ
T24
UMUC3

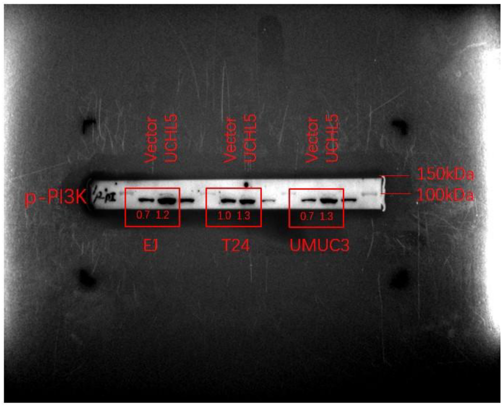
p-PI3K
Vector
UCHL5
Vector
UCHL5
Vector
UCHL5
150kDa
100kDa
EJ
T24
UMUC3

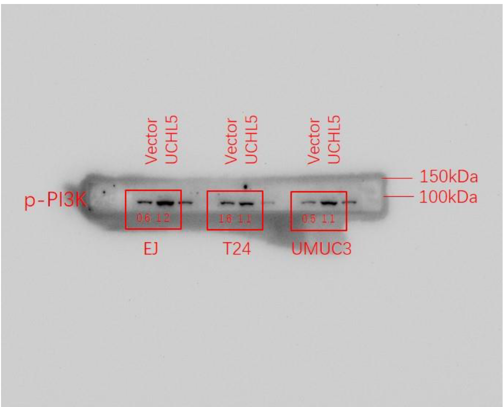
p-PI3K
Vector
UCHL5
Vector
UCHL5
Vector
UCHL5
150kDa
100kDa
EJ
T24
UMUC3

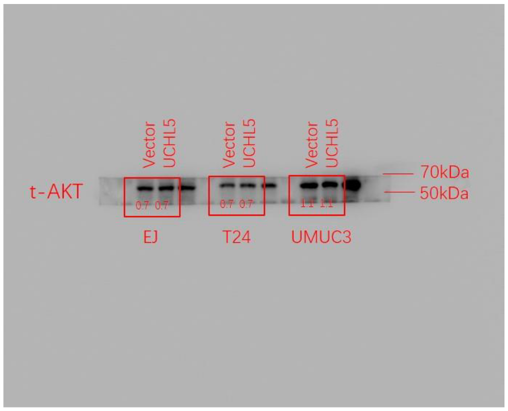
t-AKT
Vector
UCHL5
Vector
UCHL5
Vector
UCHL5
70kDa
50kDa
EJ
T24
UMUC3

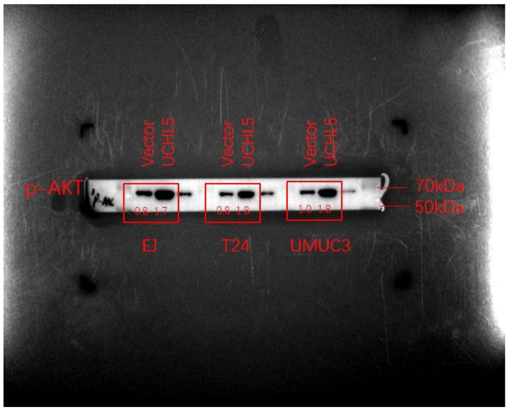
p-AKT
Vector
UCHL5
Vector
UCHL5
Vector
UCHL5
70kDa
50kDa
EJ
T24
UMUC3

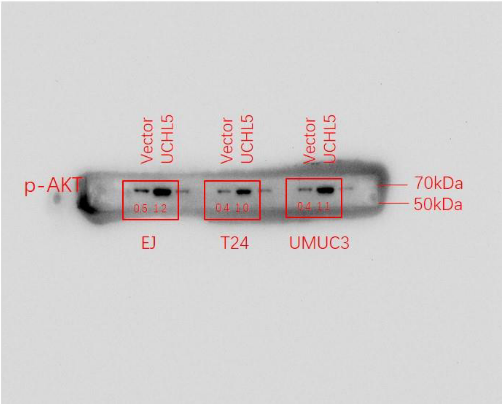
p-AKT
Vector
UCHL5
Vector
UCHL5
Vector
UCHL5
70kDa
50kDa
EJ
T24
UMUC3

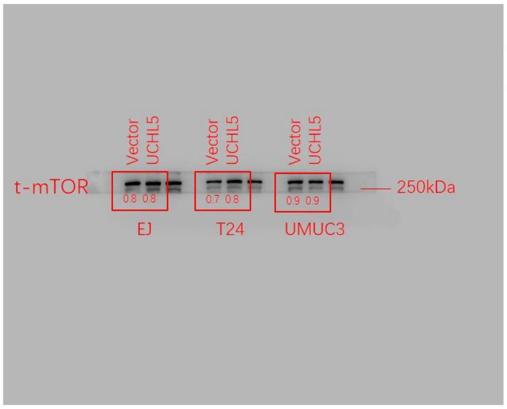


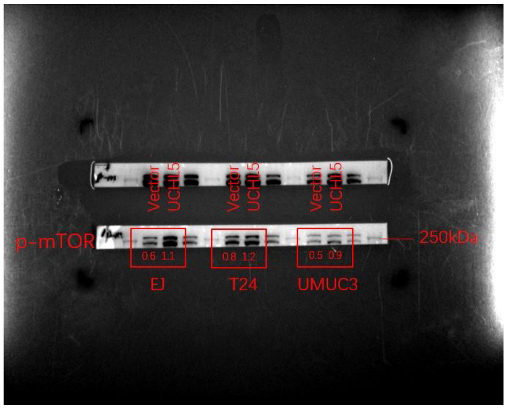


Figure 4I:  Original pictures of western blot bands including intensity ratio

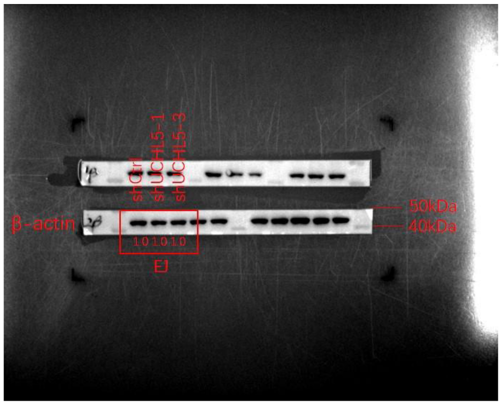


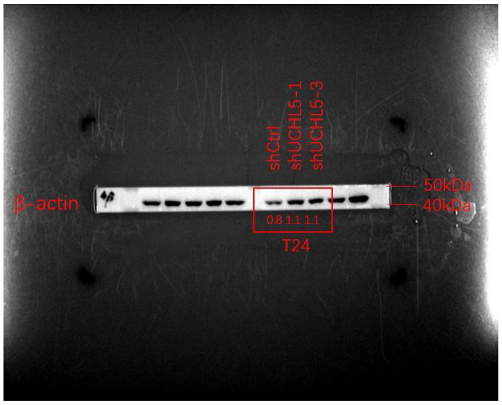


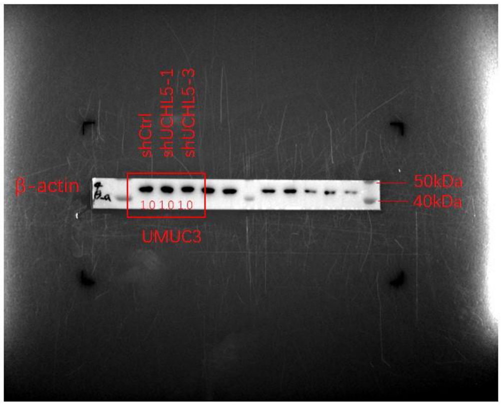


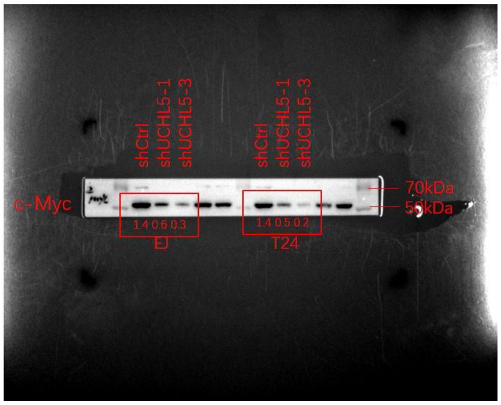

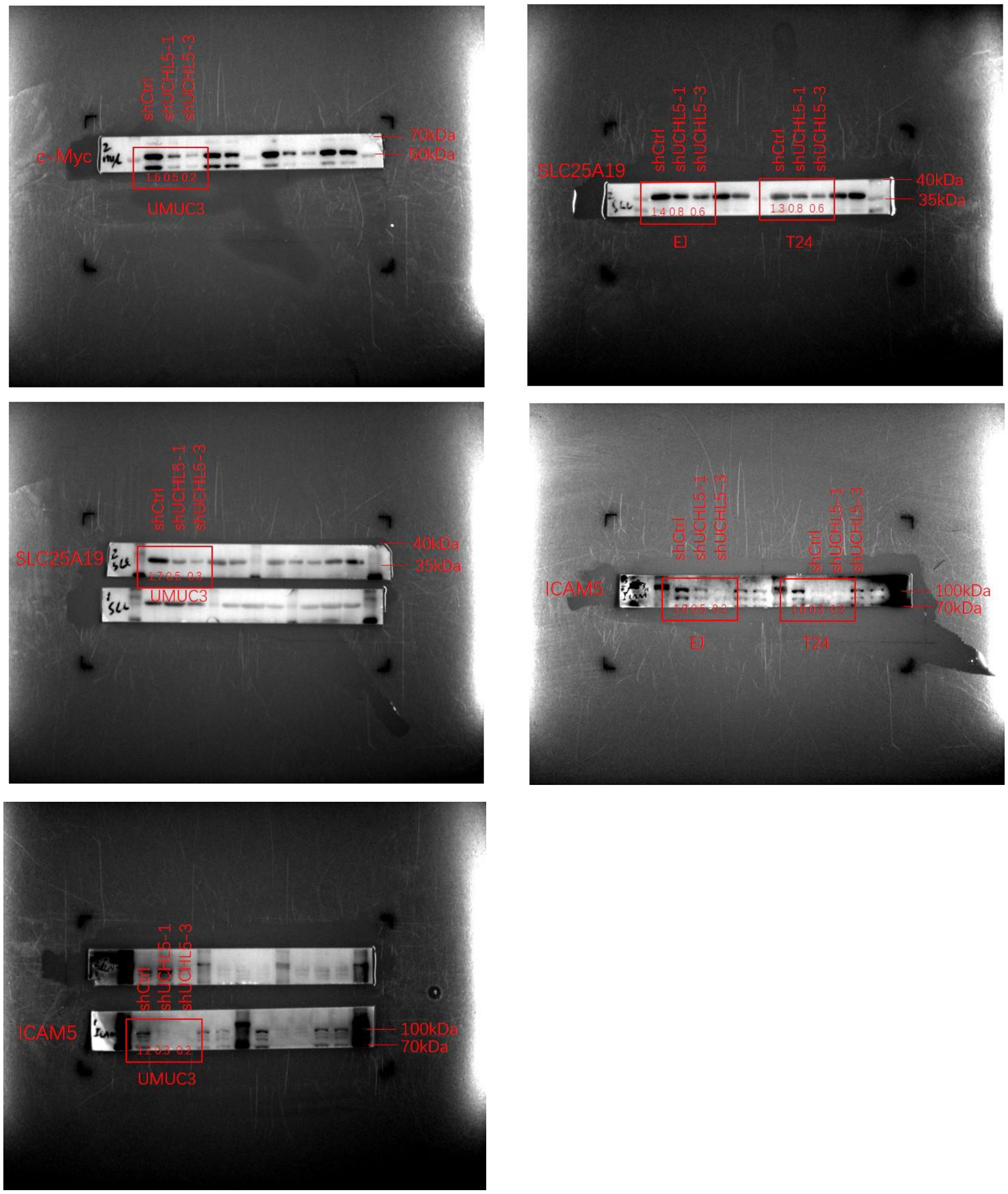


Figure 4J：Original pictures of western blot bands including intensity ratio

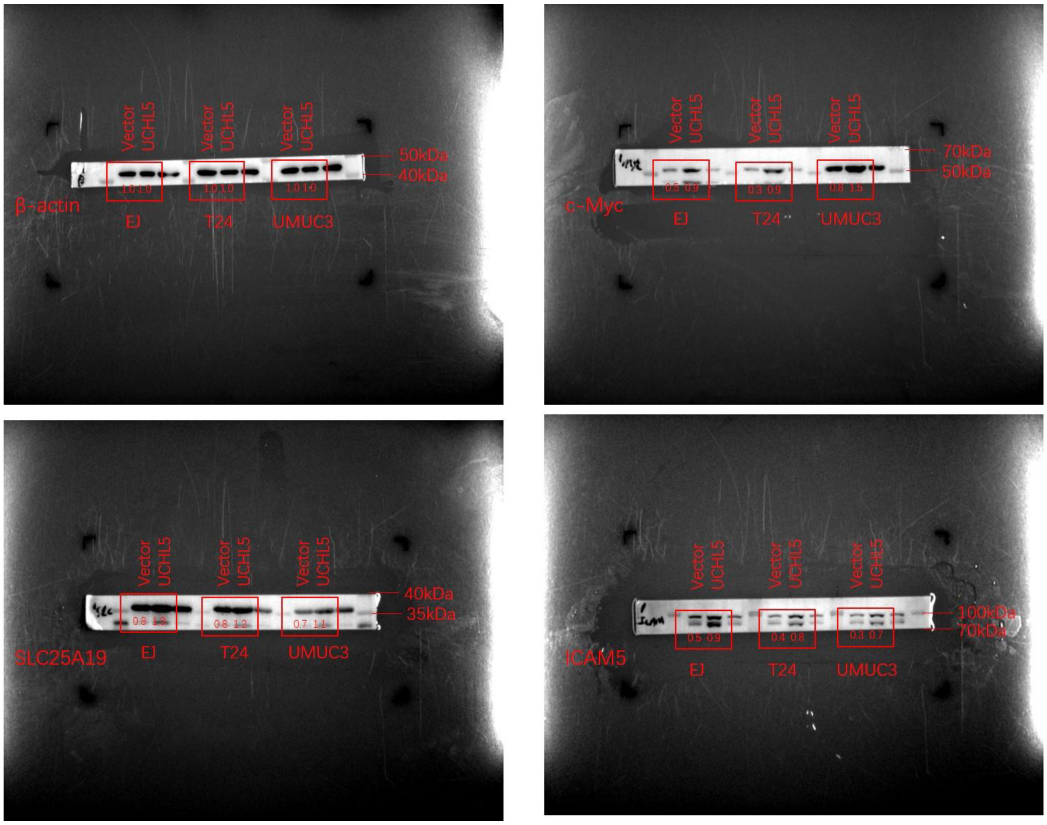


Figure 4K: Original pictures of western blot bands including intensity ratio

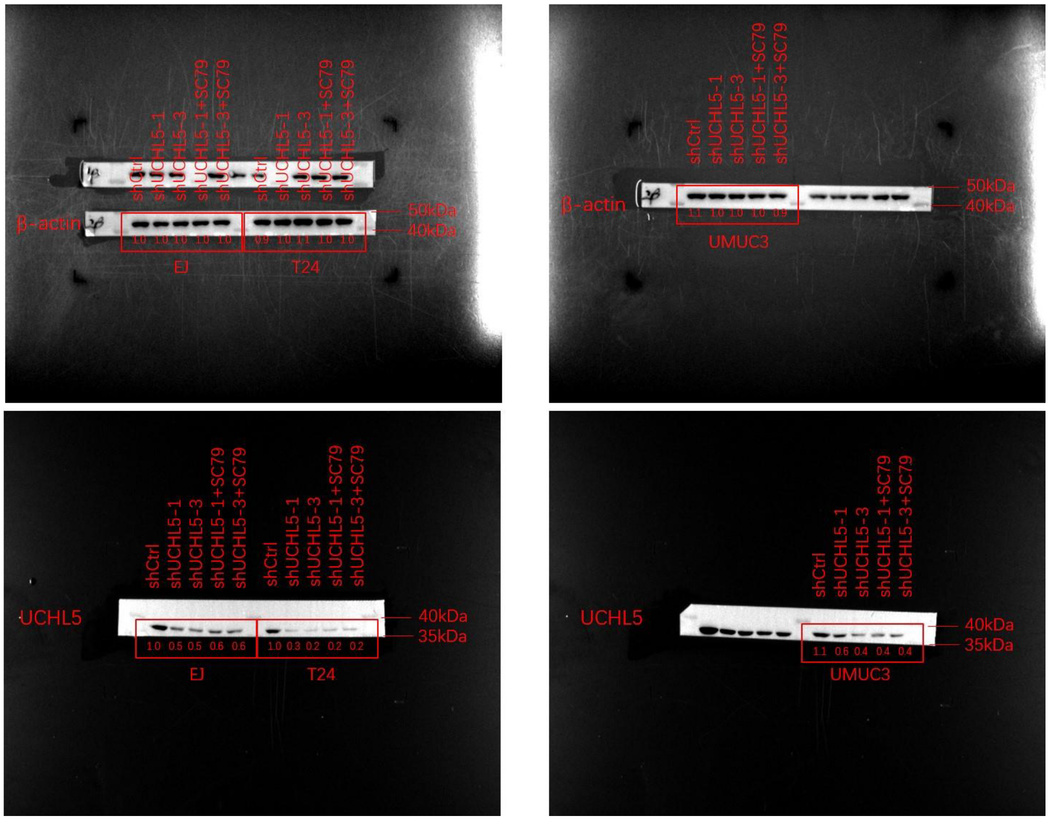

Supplement: Supplementary file 1 [file cancers-14-05538-s001.zip › cancers-1989063-origin western blots.pdf]
