# Supplementary material for: UCHL5 Promotes Proliferation and Migration of Bladder Cancer Cells by Activating c-Myc via AKT/mTOR Signaling
Source: Cancers (Basel). 2022 Nov 10;14(22):5538. doi: 10.3390/cancers14225538 (PMC9688806; doi:10.3390/cancers14225538)
Supplement: Supplementary file 1 [file cancers-14-05538-s001.zip › cancers-1989063-supplementary-PROOF.pdf]

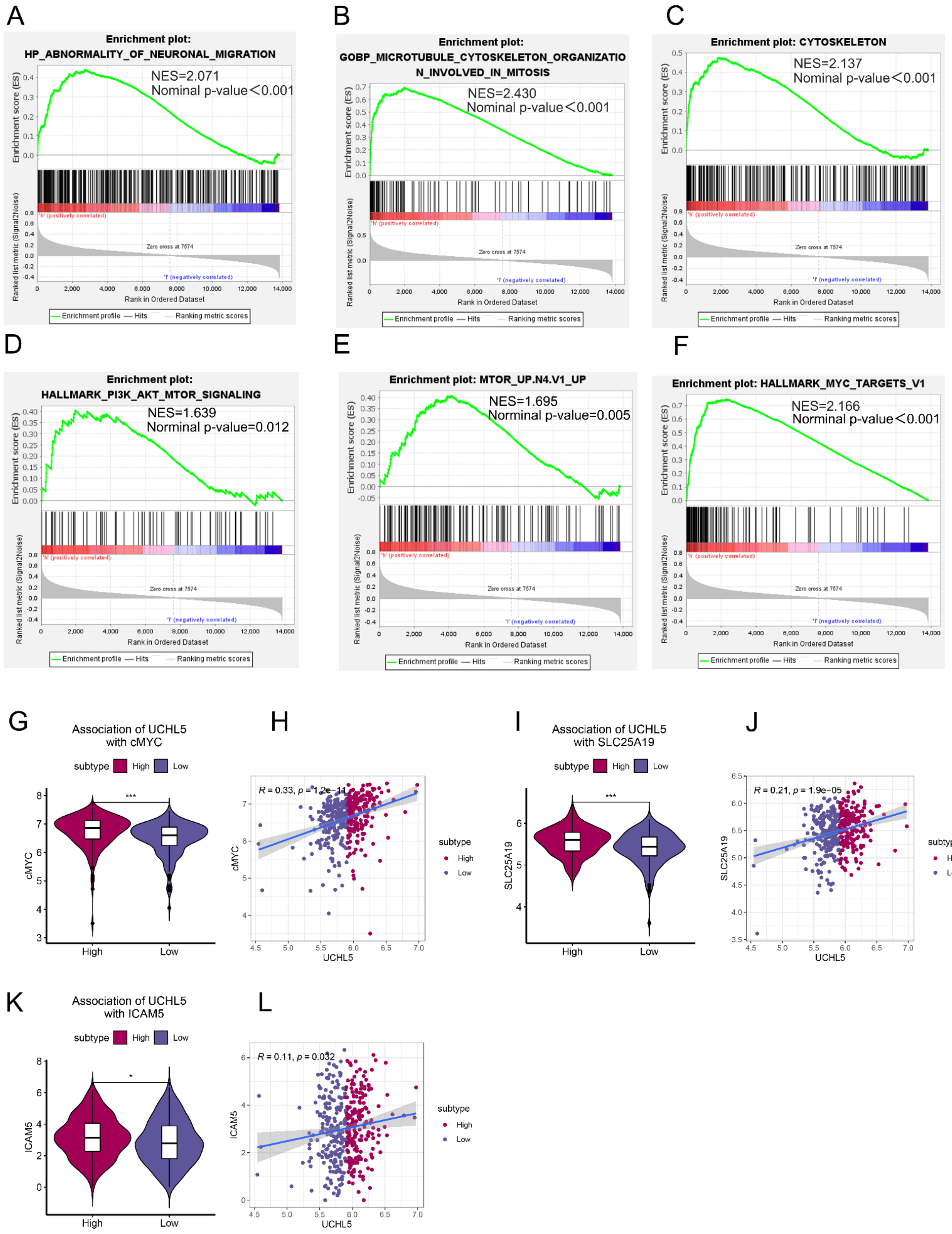

Figure S1. Bioinformatics Analysis demonstrates UCHL5 knockdown inhibits migration, cytoskeleton, and AKT/mTOR-c-Myc signaling pathway. **(A-C)** Data from RNA-seq and GSEA present that

UCHL5 knockdown represses the migration and cytoskeleton signaling pathway. **(D-F)** Data from the TCGA database show that UCHL5 knockdown inhibits the PI3K/AKT/mTOR signaling pathway and c-Myc expression. **(G)** The difference in c-Myc scores is defined using ssGSEA between the UCHL5 high- and low-expressed groups. **(H)** The association of UCHL5 expression and c-Myc score. **(I)** Difference in SLC25A19 expression between UCHL5 high - and low-expressed groups. **(J)** Association of UCHL5 and SLC25A19 expression. **(K)** Differences in ICAM5 expression between UCHL5 high- and low-expressed groups. **(L)** Association of UCHL5 and ICAM5 expression.

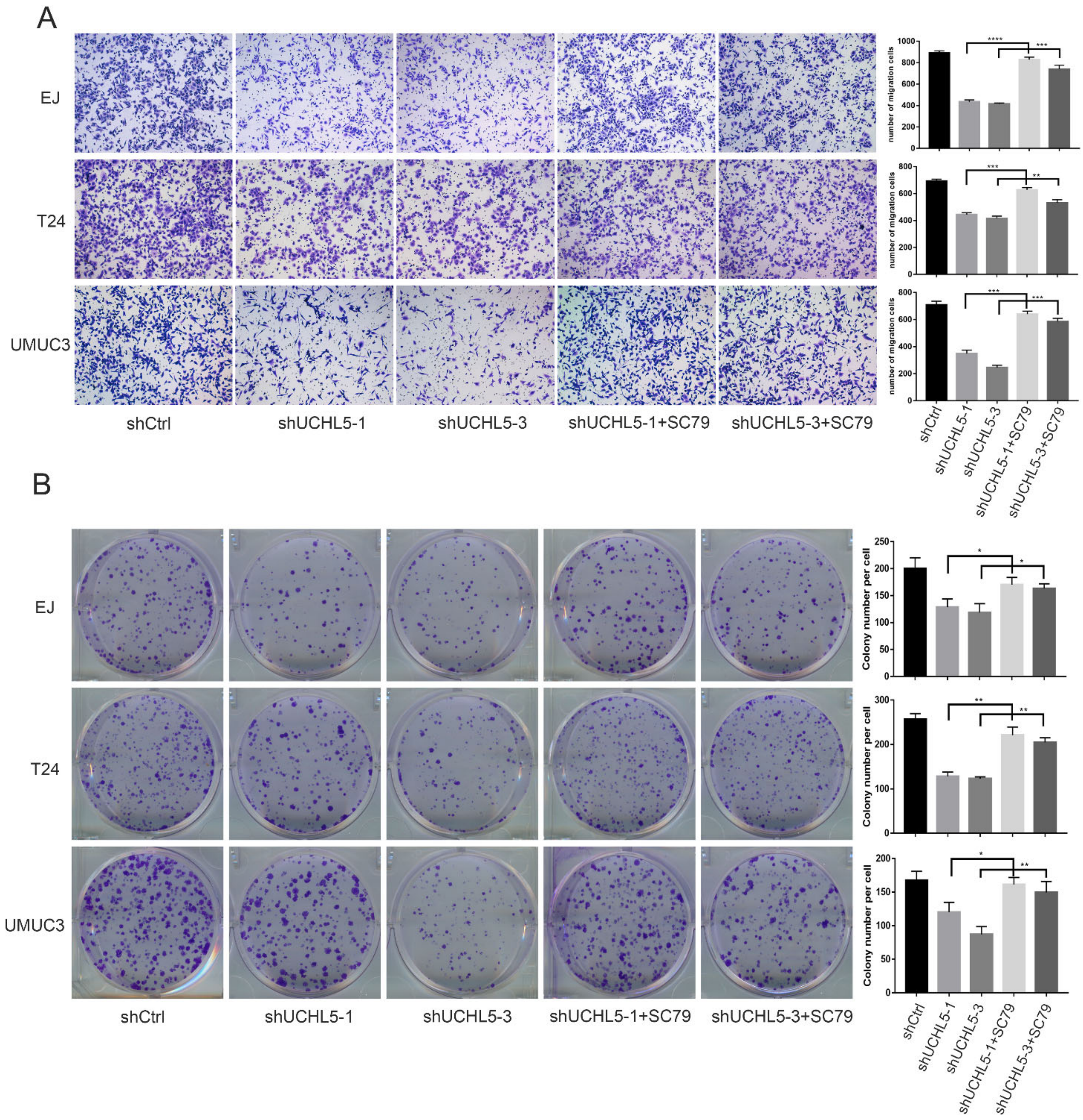

Figure S2. SC79 reverses the reffects of UCHL5 silencing in bladder cancer cells. (**A, B**) The UCHL5-1 group is removed to for increased conciseness and clarity of the images in Figure 5A,B, and the complete image with the UCHL5-1 group can be viewed in Supplementary Figure 2. We found that SC79, an activator of AKT, completely reverses the diminished cell migration and colony formation ability resulting from transfection of UCHL5-1 and UCHL5-3 shRNA.

Table S1. Primer sequences for qRT-PCR.

Gene (Human)	Forward primer (5′~3′)	Reverse primer (5′~3′)
ACTB	CATGTACGTTGCTATCCAGGC	CTCCTTAATGTCACGCACGAT
c-Myc	GGCTCCTGGCAAAAGGTCA	CTGCGTAGTTGTGCTGATGT
SLC25A19	GGTCTGTGTCTGGACTTGTTAC	GCTGAAGCTGGAAACGGATCT
ICAM5	CAGAGGGGTTTGCGTTGGTT	GAAAGTGCGAATGAGCCCAC

Table S2. Primary antibodies for Western Blot and immunohistochemical.

Antigens (Human)	Species antibodies raised in	Dilution	Supplier
UCHL5	Rabbit, polyclonal	1:1000 (WB)<br>1:200 (IHC)	Abclonal A7978
t-AKT	Rabbit, monoclonal	1:1000 (WB)	Abclonal A17909
p-AKT	Rabbit, monoclonal	1:1000 (WB)<br>1:200 (IHC)	Abclonal AP0637
t-mTOR	Rabbit, polyclonal	1:1000 (WB)	Abclonal A11354
p-mTOR	Rabbit, monoclonal	1:1000 (WB)	Abclonal AP0115
t-PI3K	Rabbit, monoclonal	1:200 (IHC)	Abclonal A19684
p-PI3K	Rabbit, polyclonal	1:1000 (WB)<br>1:200 (IHC)	Abclonal AP0854
c-Myc	Rabbit, monoclonal	1:1000 (WB)<br>1:200 (IHC)	Cell Signaling #9402
SLC25A19	Rabbit, polyclonal	1:1000 (WB)	Abclonal A12373
ICAM5	Rabbit, polyclonal	1:1000 (WB)	Abclonal A4156
Ki67	Mouse, monoclonal	1:200 (IHC)	Cell Signaling #9449
β-actin	Rabbit, monoclonal	1:1000 (WB)	Abclonal AC038

Table S3. Secondary antibodies for Western Blot and immunohistochemical.

Secondary detection system used	Species antibodies raised in	Dilution	Supplier
Anti-Rabbit-IgG (H+L)-HRP	Goat	1:10000 (WB)	Abclonal, USA, Cat. AS014
Anti-rabbit IgG H&L(HRP)	Goat	1:2000 (IHC)	Abcam, USA, cat. ab205718
Anti-mouse IgG H&L(HRP)	Goat	1:2000 (IHC)	Abcam, USA, cat. Ab678
